# Cooperation, Competition, and Specialized Metabolism in a Simplified Root Nodule Microbiome

**DOI:** 10.1128/mBio.01917-20

**Published:** 2020-08-25

**Authors:** Bridget L. Hansen, Rita de Cassia Pessotti, Monika S. Fischer, Alyssa Collins, Laila El-Hifnawi, Mira D. Liu, Matthew F. Traxler

**Affiliations:** a Department of Plant and Microbial Biology, University of California, Berkeley, Berkeley, California, USA; b Department of Molecular and Cellular Biology, University of California, Berkeley, Berkeley, California, USA; c Department of Chemistry, University of California, Berkeley, Berkeley, California, USA; Geisel School of Medicine at Dartmouth

**Keywords:** antibiotic, microbial interactions, microbiome, root nodule, specialized metabolism

## Abstract

Microbiomes associated with various plant structures often contain members with the potential to make specialized metabolites, e.g., molecules with antibacterial, antifungal, or siderophore activities. However, when and where microbes associated with plants produce specialized metabolites, and the potential role of these molecules in mediating intramicrobiome interactions, is not well understood. Root nodules of legume plants are organs devoted to hosting symbiotic bacteria that fix atmospheric nitrogen and have recently been shown to harbor a relatively simple accessory microbiome containing members with the ability to produce specialized metabolites *in vitro*. On the basis of these observations, we sought to develop a model nodule microbiome system for evaluating specialized microbial metabolism *in planta*. Starting with an inoculum derived from field-grown Medicago sativa nodules, serial passaging through gnotobiotic nodules yielded a simplified accessory community composed of four members: Brevibacillus brevis*, Paenibacillus* sp., Pantoea agglomerans, and *Pseudomonas* sp. Some members of this community exhibited clear cooperation *in planta*, while others were antagonistic and capable of disrupting cooperation between other partners. Using matrix-assisted laser desorption ionization–imaging mass spectrometry, we found that metabolites associated with individual taxa had unique distributions, indicating that some members of the nodule community were spatially segregated. Finally, we identified two families of molecules produced by B. brevis *in planta* as the antibacterial tyrocidines and a novel set of gramicidin-type molecules, which we term the britacidins. Collectively, these results indicate that in addition to nitrogen fixation, legume root nodules are likely also sites of active antimicrobial production.

## Abstract (precis)

Microbiomes associated with various plant structures often contain members with the potential to make specialized metabolites, e.g., molecules with antibacterial, antifungal, or siderophore activities. However, when and where microbes associated with plants produce specialized metabolites, and the potential role of these molecules in mediating intramicrobiome interactions, is not well understood. Root nodules of legume plants are organs devoted to hosting symbiotic bacteria that fix atmospheric nitrogen and have recently been shown to harbor a relatively simple accessory microbiome containing members with the ability to produce specialized metabolites *in vitro*.

## INTRODUCTION

Plants harbor distinct microbial communities associated with their roots (1–3), stems (2–4), leaves (2–5), and flowers (6, 7). These communities can influence host phenotypes in a variety of ways that are beneficial, including enhanced growth (6, 8), drought tolerance (2, 3, 9), and disease resistance (2, 3, 6). The content of these microbiomes is thought to be shaped by a combination of historical contingency (i.e., the order of arrival of strains) (10), microbial interspecies interactions (11, 12), and nutrients or other compounds exuded by the plants themselves that may select for microbes with beneficial characteristics (13, 14). Specialized metabolites produced by microbes, including molecules with antibacterial, antifungal, and siderophore activities, have been hypothesized to play a role in shaping plant microbiomes. For example, specialized metabolites may influence interactions between members of plant microbiomes (15–17) and may be a mechanism of protection from pathogen invasion (7, 17). Thus, there is interest in leveraging specialized metabolism by plant-associated microbes in agriculture and for discovery of novel compounds.

Multiple reports have demonstrated that plant microbiomes contain members with strong potential as sources of novel specialized metabolites *in vitro* (2, 15, 16, 18, 19); however, relatively few studies have examined microbial specialized metabolism *in planta* (17, 20–32). Members of the genus *Pseudomonas* and the order *Rhizobiales* are notable exceptions, as genetic approaches have been used in these organisms to demonstrate the effect of specialized metabolites which inhibited fungal pathogens (33, 34) and mediated microbe/host plant communication (35, 36), respectively. From a chemical perspective, our knowledge of specialized metabolism *in planta* is much more limited, with only a few reports of demonstrations of detection of antimicrobials *in planta* (37–40). Thus, while specialized metabolisms appear to be widespread in plant microbiomes, many questions remain regarding when and where these molecules are produced *in planta* and what their impact may be within these microbial communities.

Legume plants are notable from a microbial perspective because they form specialized, N-fixing organs, called nodules, through intimate association with bacterial symbionts of the orders *Rhizobiales* and *Burkholderiales*. Biological N-fixation by legumes plays a significant role in the global N cycle, with estimates of N fixed per year on a global scale ranging from 39 to 70 Gg (41, 42). Owing to the agricultural and ecological importance of N-fixation, this plant-microbe symbiosis has been the subject of intense research for several decades (35, 43–53). As a result, much is known about the genes and chemical signals and molecular mechanisms that underpin this symbiosis (35, 54–56). While nodulation has traditionally been studied as a two-member system, it has more recently become clear that root nodules harbor an accessory microbiome (18, 57–60). In a recent study, Xiao and coworkers found that the different rhizocompartments of Medicago sativa (i.e., the rhizosphere, root endosphere, and nodules) were successively limited in microbial diversity, with the nodule containing the simplest community (57).

Several lines of *in vitro* evidence suggest that members of the nodule microbiome may be rich sources of specialized metabolites. For example, a novel antibiotic, phazolicin, from a *Rhizobium* sp., isolated from the root nodules of Phaseolus vulgaris (wild beans) was previously described (61). Additionally, *Micromonospora* and *Paenibacillus* sp. isolated from the root nodules of M. sativa, showed antifungal activity against common phytopathogens *in vitro* (18, 62–64). The relative simplicity of the nodule microbiome and the observation that members of this microbiome have potential for specialized metabolism make the nodule an attractive system for exploring microbial interactions and the ecological roles of specialized metabolites *in situ*. With this in mind, we sought to develop a tractable nodule microbiome system (i) whose members were derived from field-grown plants, (ii) that was easily experimentally manipulated, and (iii) that enabled interrogation of the system at the chemical level. Here, using a combination of community profiling and a simplified nodule community, we report that the M. sativa root nodule microbiome is dynamic over time and life phase, that the nodule microbiome contains members that strongly interact through cooperation and competition, and that microbes within the nodule community produce specialized metabolites, including a novel antimicrobial, *in planta*. Taken together, these results lend support to the idea that in addition to nitrogen fixation, legume root nodules are sites of active antimicrobial production.

## RESULTS

### Community profiling across root nodule development.

Here, we sought to develop an experimentally tractable root nodule microbiome system using *Medicago sativa* (alfalfa). This legume was chosen since protocols are readily available for its gnotobiotic germination and nodulation (65) and the plants are small enough to enable good scalability for experiments requiring many replicates. Beyond this, alfalfa is of interest from an agriculture perspective, being the fourth most widely grown crop in the United States (66).

**FIG 1:**
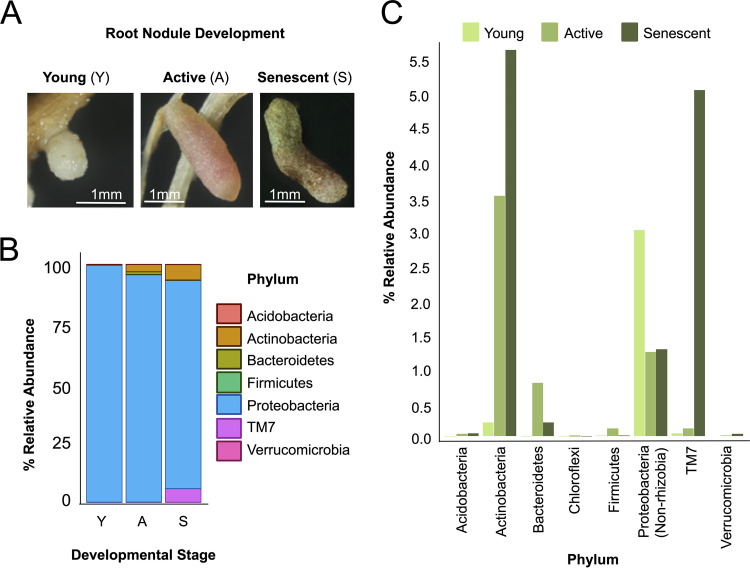
16S community profiling of three different root nodule developmental phenotypes from agricultural M. sativa plants. (A) Images of young (Y, white), active (A, pink/red), and senescent (S, brown/green) nodules. (B) Relative abundance of each phylum based on 16S amplicon sequencing of young, active, and senescent root nodules from an agricultural field in Alturas, CA. (C) Relative abundance of each phylum, excluding *Rhizobia*.

Over the last decade, non-*Rhizobiales* members of nodule microbiomes have previously been detected in root nodule tissue (57). However, it is unclear if these bacteria are present across the lifetime of individual nodules. We define the following three developmental phases of the alfalfa root nodule: young nodules (small and white), active nodules (pink/red), and senescent nodules (brown/green coloration) (Fig. 1A). The pink color in active nodules is due to the presence of leghemoglobin, whereas the brown/green color present during senescence is due to the degradation of the heme group associated with leghemoglobin (67). To address whether or not the nodule microbiomes differed across these phases, we harvested nodules from established alfalfa plants (∼10 years) from an agricultural field in Alturas, CA. We classified each nodule as young, active, or senescent, extensively washed its outer surface, and extracted DNA for 16S amplicon sequencing (SRA accession no. PRJNA608732).

We found that an accessory community was present throughout the nodule life cycle (Fig. 1B) and that the relative abundances of this community differed between developmental stages. For instance, *Ensifer* (*Proteobacteria*) made up 94% to 96% of the operational taxonomic units (OTUs) in young nodules and active nodules, but its relative abundance was reduced to ∼86%, in senescent nodules. Representatives of the genus *Pseudomonas* had an average relative abundance of 2.45% in young nodules; however, their levels decreased to 0.03% and 0.05% in active and senescent nodules, respectively. In contrast, the relative abundances of actinobacterial taxa *Streptomyces*, *Actinoplanes*, and *Micromonosporaceae*_unclassified all increased from ∼0.1% in young nodules to ∼1.60% in senescent nodules. TM7 is a phylum thought to be associated with *Actinobacteria*, and it also increased in relative abundance across these developmental stages from 0.06% to 0.10% and 5.70%. Similar relative abundance changes captured across the processes of root nodule development were observed in M. sativa grown under various soil conditions (see Fig. S1 at https://doi.org/10.6084/m9.figshare.12107094, where all of the supplemental items can be found). Collectively, these results indicate that the root nodule microbiome is both simple and dynamic as nodules mature from the young phase through that active and senescent phases.

### Root nodule microbial community selection.

**FIG 2:**
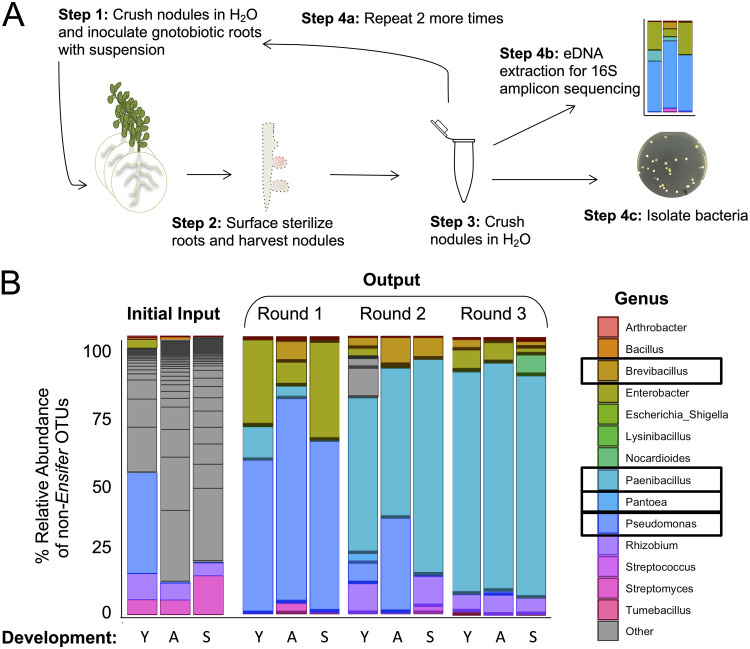
Root nodule microbial community selection with M. sativa. (A) Schematic showing the workflow for creating the microbial community. (Step 1) Crushed nodules were inoculated onto gnotobiotic plant roots. Plants were incubated until nodules formed. (Step 2) Roots were then surface sterilized and nodules removed and sorted based on phenotype. (Step 3) Nodules were then crushed and resuspended in water. (Step 4) This homogenous mixture was used for three applications: for repeating the process by application to gnotobiotic roots (Step 4a), for environmental DNA extraction for 16S amplicon sequencing (Step 4b), and for isolation of bacteria (Step 4c). (B) Relative abundances of non-*Ensifer* operational taxonomic units (OTUs) at the genus level from each successive root nodule inoculum (“Round”) across the root nodule phenotypes. Black boxes indicate genera isolated after round 3 and used for subsequent experiments.

The agricultural root nodule microbiome has been described as having considerably less richness than the root and surrounding bulk soil (57, 58, 68, 69). This simplicity prompted us to consider if we could develop an experimentally tractable nodule microbiome system (70). In order to do this, we used *in planta* selection to arrive at a simplified bacterial community. We started with agricultural nodules from all three developmental phases, surface sterilized them, and homogenized them to obtain a comprehensive inoculum. This homogenized material was resuspended in sterile water and inoculated onto the roots of gnotobiotic 3-day-old M. sativa seedlings. These seedlings were also inoculated with Sinorhizobium meliloti RM1021, the well-studied, nodulating symbiont of M. sativa. We grew these plants under controlled conditions for 3 to 5 weeks and harvested root nodules from all three phenotypic stages for parallel 16S community profiling (SRA accession no. PRJNA608732) and bacterial isolations. Nodules from all three phenotypic stages were once again surface sterilized and homogenized to obtain the inoculum for the next passaging round (Fig. 2A). We repeated these steps three times and arrived at a final simplified community comprised of the four accessory bacterial members Brevibacillus brevis Ag35, *Paenibacillus* sp. Ag47, *Pseudomonas* sp. Ag54, and Pantoea agglomerans Ag15, plus the nodulating strain Sinorhizobium meliloti RM1021 (Fig. 2B; see also Table S1 at https://doi.org/10.6084/m9.figshare.12107094).

Figure 2B shows that the four members of this community rose to prominence in the accessory microbiome relatively quickly during the first round of passaging and continued to dominate the accessory community through the second and third passages. These data indicate that this accessory community was the product of relatively strong selection in our gnotobiotic nodule system. We note that OTUs representing each of the four accessory members of this community were detected in our initial 16S community profile from field-grown nodules and that members of each of these taxa have been found as members of the nodule microbiome in other studies (18, 57, 71). Inoculation with this community did not affect plant height or nodule number relative to control plants (see Fig. S2A and B).

### *In planta* community assembly and recoverability.

**FIG 3:**
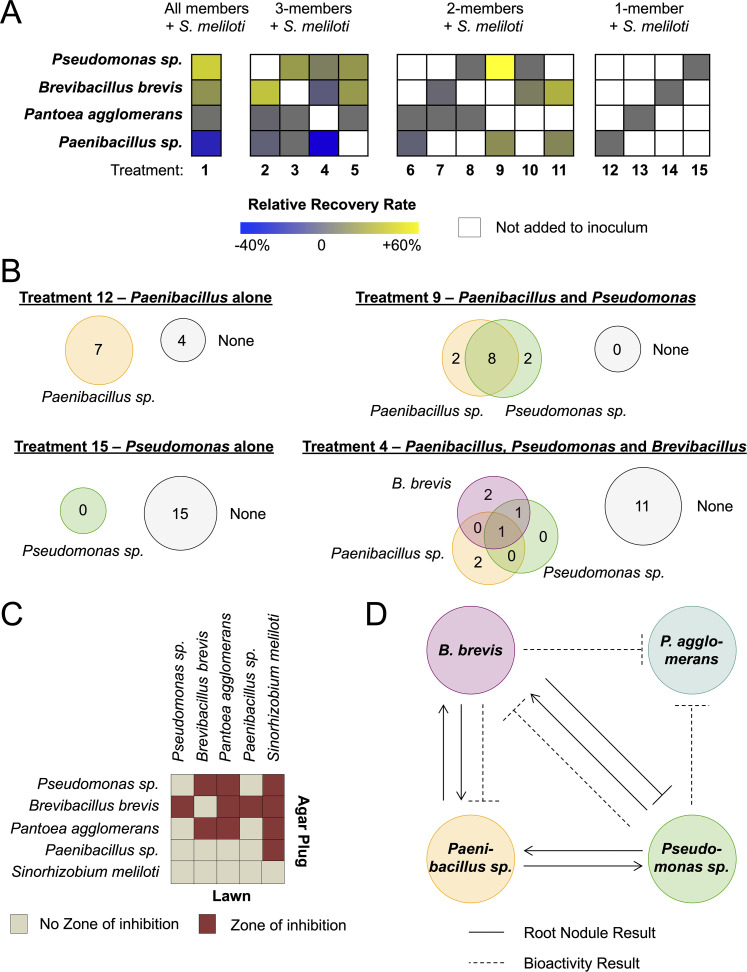
Interactions between members of the synthetic root nodule community. (A) Rate of reisolation of (or recovery) each bacterium from M. sativa roots that were inoculated with the bacterial isolates that resulted from the selection process detailed in Fig. 2. Experimental treatments were all possible combinations of these bacteria inoculated onto M. sativa roots with the essential nodulation strain, S. meliloti. These plants were grown until they developed root active nodules, and then bacteria were systematically reisolated from these root nodules. Recovery rates for each bacterium are relative to the recovery rate from plants that were inoculated with one bacterium plus S. meliloti. White boxes indicate that no bacterium was added to the inoculum. Gray, zero change; blue, negative relative recovery; yellow, positive relative recovery. (B) The number of bacterial colonies recovered from treatments 12, 9, 15, and 4 (see Fig. S3 for all other treatments). Numbers within gray circles labeled “None” represent the number of nodules where no bacterium was recovered, while numbers in colored circles represent the number of nodules where one or more bacteria were recovered. (C) Bioactivity agar plug diffusion assay of each member against a lawn of each member on root nodule agar medium. Red squares indicate that a zone of inhibition was observed, and tan squares indicate that no inhibition zone was observed. (D) Model summarizing the interactions observed for microbe recovery *in planta* (A and B) and *in vitro* (C).

Given that the relative abundance measurements presented in Fig. 2B represent the average community across 10 nodules, we sought to assess colonization/maintenance of the accessory community at the level of individual nodules. To do so, we combined the gnotobiotic alfalfa system with a culture-based approach to assess the recovery rate (i.e., the percentage of nodules containing a given bacterial strain) of each community member. First, we inoculated each microbe alone on 3-day-old seedling roots and found that each microbe was insufficient to generate nodules (see Fig. S2), as expected. We next set up a series of experiments in which each microbe was coinoculated with the nodulating strain, S. meliloti RM1021. For these accessory community experiments, unless otherwise noted, S. meliloti RM1021 was always included. Two of the four accessory community members were recoverable from active root nodules when they represented the sole accessory community member, i.e., Brevibacillus brevis Ag35 (recovery rate of 40%) and *Paenibacillus* sp. Ag47 (64%). The individual recovery rates were highly variable for each microbe under conditions of coinoculation in combination with other accessory members, as summarized in Fig. 3A (see also Table S2 and S3).

Within these combinations, examples of ecological cooperation and competition, as defined by Mitri and Foster (72), were apparent (Fig. 3B; see also Fig. S3 and Table S6). For example, *Pseudomonas* sp. Ag54 was unrecoverable (0%) when it was the sole accessory community member in all 15 nodules assayed (Fig. 3B). However, when we added *Paenibacillus* sp. Ag47 and *Pseudomonas* sp. Ag54 (together with S. meliloti), we observed that each member’s recovery rate increased to 83% (Fig. 3A) and that 8 of 12 nodules contained both members (Fig. 3B), with zero nodules lacking at least one of these strains. This represented a 20% increase for *Paenibacillus* sp. Ag47 and a striking 83% increase for *Pseudomonas* sp. Ag54. This positive interaction changed when we added B. brevis Ag35 (highlighted in Fig. 3B). When all three microbes where included, the recovery rates of *Paenibacillus* sp. Ag47 and *Pseudomonas* sp. Ag54 decreased by ∼66% and ∼72%, respectively, compared to the results seen when they were inoculated as a pair. Thus, the addition of B. brevis Ag35 abrogated the observed benefits of cooperation between *Pseudomonas* sp. Ag54 and *Paenibacillus* sp. Ag47. This negative impact indicates that B. brevis Ag35 shifted the community into a mode driven by competition rather than by cooperation.

We noted that whereas the observed cooperation between *Pseudomonas* sp. Ag54 and *Paenibacillus* sp. Ag47 was evident in terms of recovery rate, in terms of the abundance of each partner, i.e., the CFU counts, the results were complex (see Table S6 and Fig. S17). *Pseudomonas* sp. Ag54 was unrecoverable (0 CFU/nodule) under conditions of inoculation with S. meliloti RM1021 alone; however, when coinoculated with *Paenibacillus* sp. Ag47, the average count increased to ∼65 CFU/nodule. Interestingly, when *Paenibacillus* sp. Ag47 was coinoculated with only S. meliloti RM1021, it had an average CFU count of ∼280/nodule; however, when coinoculated with *Pseudomonas* sp. Ag54, this number decreased by ∼4-fold (to ∼69 CFU/nodule). This negative effect was unexpected given the enhanced recoverability of *Paenibacillus* sp. Ag47 seen with the addition of *Pseudomonas* sp. Ag54. These data indicate that microbial interactions can have disparate effects on different aspects of life in root nodules. In this example, *Pseudomonas* sp. Ag54 and *Paenibacillus* sp. Ag47 may be cooperative during the nodule colonization process (affecting recoverability) and yet the combination may result in a cost for *Paenibacillus* sp. Ag47 at the level of average abundance over time.

### Bioactivity of accessory community members.

Several studies have revealed that bacteria isolated from root nodule tissue can frequently make bioactive compounds *in vitro* (See reference 18 for a comprehensive review). Therefore, we hypothesized that the outcomes that we observed in our *in planta* experiments could have been due to antibiotic-mediated interspecies interactions. To address this possibility, we grew each microbe on medium designed to mimic nutrients available within nodules (described in Materials and Methods; here referred to as “root nodule medium”) and assayed each microbe’s inhibitory activity against lawns of all the other community members. We found that all accessory members had inhibitory activity against one or more members and that all of the members inhibited S. meliloti RM1021 growth (Fig. 3C). We used the outcome of this assay in tandem with the outcomes of the *in planta* experiments to produce a summary map for interactions within this simplified community (Fig. 3D).

### Specialized metabolism *in planta*.

**FIG 4:**
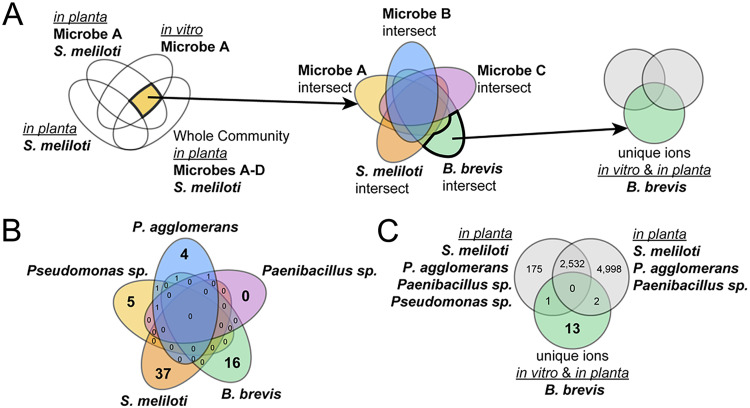
Number of molecular features associated with the simplified nodule community *in vitro* and *in planta*. Molecular features identified via LC/MS from chemical extracts that were prepared from one of the three independent treatments of each microbe (present alone on agar plates [*in vitro*], inoculated onto M. sativa plants individually with S. meliloti, or inoculated onto M. sativa plants with all other members of the simplified nodule community) plus an additional control treatment of M. sativa plants inoculated solely with S. meliloti. (A) Analysis approach using nested Venn diagrams to identify features that are unique to each microbe followed by focusing on unique features associated with B. brevis compared to all other community members both *in planta* and *in vitro*. (B) Numbers of features that are unique to each microbe. (C) Numbers of features that are unique to B. brevis compared with the number of features identified in two different simplified communities lacking B. brevis *in planta.* The 13 features that are unique to B. brevis *in vitro* and *in planta* are highlighted as features of interest and detailed in Table S4.

To identify molecules/unique chemical features that could serve as signatures for each of the microbes in the community, we conducted a Venn analysis with the processed metabolomics data, as seen in Fig. 4A. We identified chemical features that were not present in nodules containing only S. meliloti but that were present in all three other treatments: (i) a single microbe grown *in vitro*, (ii) the root nodule inoculated with this microbe, and (iii) the root nodule inoculated with the whole community (Fig. 4A; see also Fig. S4). Each set of molecular features present in this intersection of treatments is unique to each microbe and also present in the root nodule inoculated with the whole community. To identify chemical features that were unique to each microbe compared to the other members of the community, we performed a second Venn analysis with the outputs of the first set of analyses (Fig. 4A). We found zero unique features attributable to *Paenibacillus* sp. Ag47, 4 unique features for P. agglomerans Ag15, 5 unique features for *Pseudomonas* sp. Ag54, and 16 unique features for B. brevis Ag35 (Fig. 4B). S. meliloti had the greatest number of unique features among the five bacteria, with 37 features.

Since B. brevis Ag35 was the accessory community member that had the greatest number of unique features *in planta* and displayed the ability to inhibit growth of all other members of the community (Fig. 3C), we concluded that B. brevis Ag35 might be a promising candidate for further exploration of specialized metabolism within the root nodule. We started with the set of 16 unique chemical features from the second analysis for this microbe and compared it to features observed from two root nodule communities that did not include B. brevis Ag35. Specifically, the two communities used for this comparison were the entire community, except B. brevis Ag35, and a community consisting of S. meliloti RM1021, P. agglomerans Ag15, and *Paenibacillus* sp. Ag47. This analysis revealed 13 features that were unique to B. brevis Ag35 (Fig. 4C; see also Table S4).

### MALDI-IMS of the simplified community root nodule.

**FIG 5:**
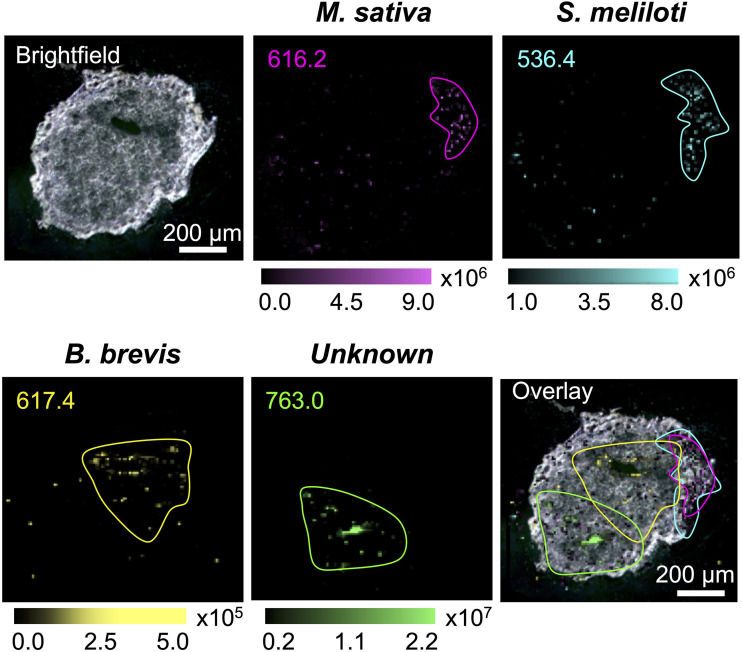
MALDI images of the simplified community root nodule. A bright-field image is shown of a 20-μm-thick simplified community root nodule embedded in gelatin. The ion for heme B, *m*/*z* 616.2 (magenta), and an unidentified feature, *m*/*z* 536.4 (cyan), are unique to S. meliloti and colocalize. In contrast, the feature *m*/*z* 617.4 is associated with B. brevis, and an unidentified feature associated with the community, *m*/*z* 763.0, is spatially distinct (Overlay).

Since we identified several multiple-microbe-specific chemical features, we sought to leverage these data to examine the distributions of species-specific chemical features/ions within nodule tissues. Detection of microbe-specific features *in planta* might allow these features to be aligned with key plant physiological processes and/or might shed light on the distribution of each microbe. We employed high-resolution matrix-assisted laser desorption ionization–imaging mass spectrometry (MALDI-IMS), which provides a two-dimensional map of molecular features in a sample. We applied this method to the surface of a 20-μm-thick slice from an active root nodule that was inoculated with the whole microbial community, with a pixel size of 10 μm (Fig. 5; see also Fig. S16). We observed a feature with a *m*/*z* value of 616.2 that was localized in an area proximal to the site of stem attachment. This ion (*m*/*z* 616.2) had a mass and fragmentation pattern (observed by liquid chromatography-tandem mass spectrometry [LC-MS/MS]) that exactly matched published data for the identification of heme B (73) and a protoporphyrin standard (see Fig. S5 and Table S5). Thus, we ascribe this feature to heme B associated with leghemoglobin produced by M. sativa within the nodule region where active nitrogen fixation would be expected.

On the basis of the analysis represented in Fig. 4, we next checked for features that were diagnostic for each of the microbial members of the system. A feature associated exclusively with S. meliloti RM1021, *m*/*z* 536.4, showed strong colocalization with the heme ion noted above. This colocalization was significant and expected because it is consistent with the presence of S. meliloti RM1021 in the region of nitrogen fixation. We also observed that a feature unique to B. brevis Ag35, *m*/*z* 617.4, was detected in the central area of the nodule, with little or no overlapping of the S. meliloti RM1021 feature *m*/*z* 536.4. Finally, we observed a feature with *m*/*z* of 763.0 which was uniquely associated with the entire community, meaning that this feature was detected only in nodules that were inoculated with all members of our simplified nodule microbiome. This feature was localized in the distal part of the root nodule, in an area distinct from the features associated with S. meliloti RM1021 and B. brevis Ag35. Taken together, these data demonstrate that features associated with at least two members of this simplified community, and possibly more, are detectable in different regions of the nodule, indicating that these microbes may reside in distinct locations *in situ.*

### Identification of Brevibacillus brevis Ag35 secondary metabolites.

**FIG 6:**
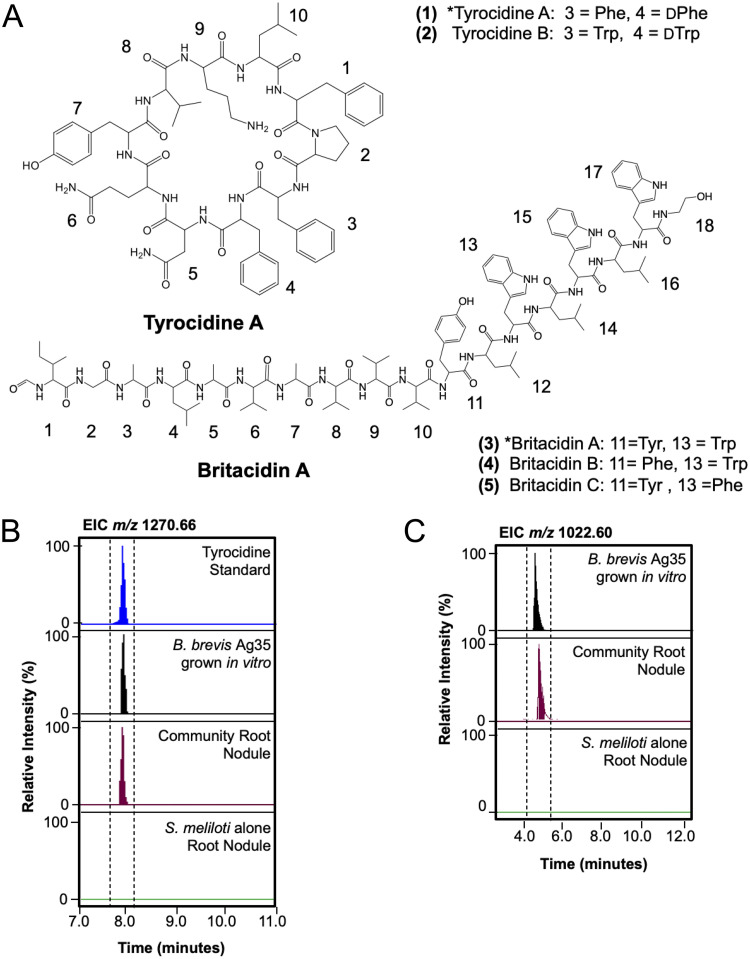
Tyrocidine A and britacidin A are detected *in planta*. (A) Tyrocidine A (amino acid position 1) and B (position 2) and britacidin A (position 3), B (position 4), and C (position 5) are produced by B. brevis. The asterisk (*) denotes that the indicated molecule was detected *in planta* (tyrocidine A and britacidin A). (B) Extracted ion chromatogram of the [M+H]^+^ tyrocidine A, *m*/*z* 1,270.66, from tyrocidine standard, B. brevis grown *in vitro* on root nodule medium, methanol extracts of community root nodules, and methanol extracts of S. meliloti-only root nodules. (C) Extracted ion chromatogram of the dominant isotope of the [M + 2H]^2+^ species of britacidin A, *m*/*z* 1,022.60, from B. brevis grown *in vitro* on root nodule medium, methanol extracts of community root nodules, and methanol extracts of S. meliloti-only root nodules.

Because we were able to detect B. brevis Ag35 features *in planta* using both LC/MS and MALDI-IMS methods, we focused on identifying some of the features observed in the metabolomics data (Fig. 4). Of the 13 ions specific to B. brevis Ag35 found in the root nodule extracts, we identified *m*/*z* 1,270.66 as the [M+H]^+^ adduct of tyrocidine A (1), a nonribosomal peptide (NRP). We verified this identification by comparing its exact mass, retention time, and fragmentation pattern to an authentic standard of tyrocidine (Fig. 6B; see also Fig. S6 and Table S5). We also found *m*/*z* 635.83, representing the [M + 2H]^2+^ ion of tyrocidine A (1). Further analysis of extracts from B. brevis Ag35 grown *in vitro* revealed that this strain also produced tyrocidine B (2), indicated in Fig. 6A (see also Fig. S7 and Table S5).

**FIG 7:**
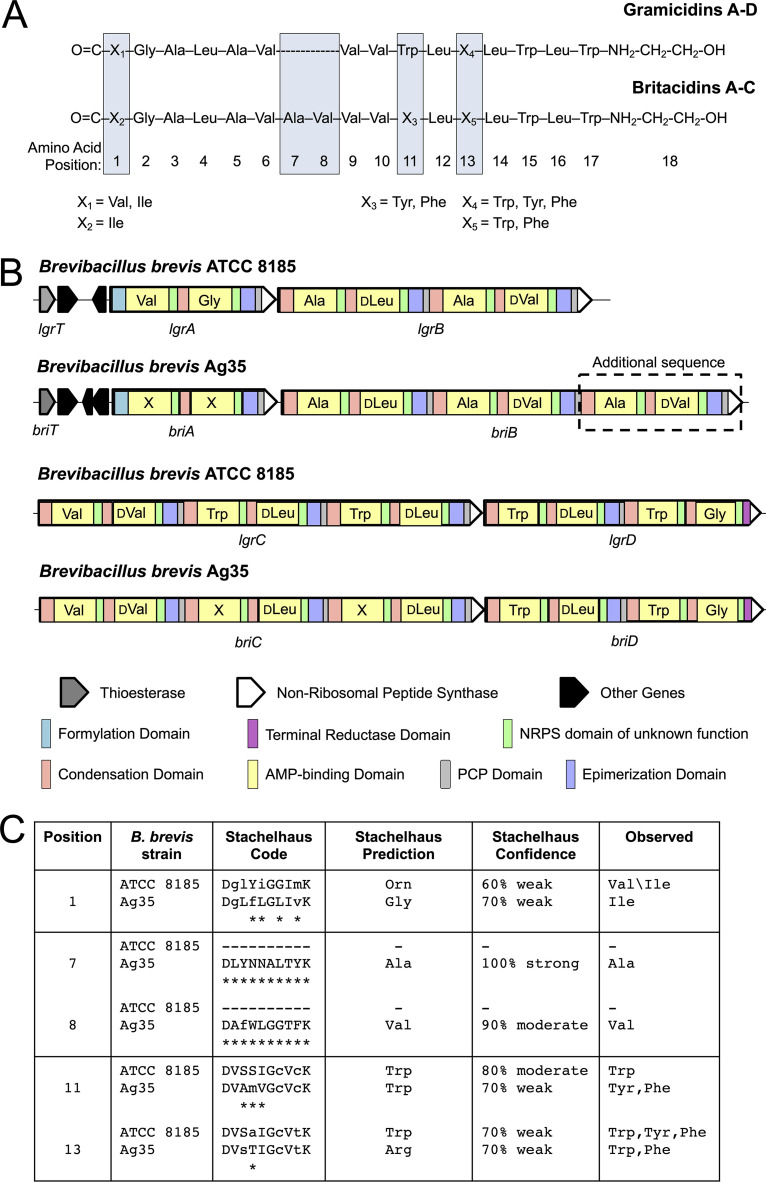
Britacidin and gramicidin comparison at the chemical and genetic levels. (A) Chemical structure comparison of britacidin A to C and gramicidin A to D, with blue boxes highlighting the structural differences. (B) Comparison of gramicidin NRPS genes *lgrA* to *lgrD* (*lgrA-D*) from B. brevis ATCC 8185 and the britacidin NRPS genes *briA-D* from B. brevis Ag35. Amino acids incorporated by the AMP-binding domains (yellow) are listed within the gene described for *LgrA-D*. Stachelhaus alignments to genes *LgrA-D* were used for amino acid assignments for nonribosomal peptide-synthetase (NRPS)*briA-D*. (C) Table highlighting chemical positions 1, 7, 8, 11, and 13 (boxed in Fig. 7A) and their respective AMP-binding domain Stachelhaus codes, alignments, predictions, and observed amino acids.

Among the features associated with B. brevis Ag35 *in planta*, we observed another ion, *m*/*z* 1,033.08 (Table S4), that had an MS1 pattern (see Fig. S8) indicative of a doubly charged species and a fragmentation pattern similar to that of the gramicidin-family compounds (see Fig. S9). The gramicidins are antibiotic NRPs known to be produced by B. brevis ATCC 8185 (74). Further analysis of *m*/*z* 1,033.08 revealed it to be the [M+H+Na]^2+^ species of a novel molecule we have termed britacidin A (3). Beyond this, we note that we also detected the [M + 2H]^2+^ (*m*/*z* 1,022.0960), [M+H+Na]^2+^ (*m*/*z* 1,033.0848), and [M + 2Na]^2+^ (*m*/*z* 1,044.0746) adducts of this compound in extracts from root nodules inoculated with the community containing Brevibacillus brevis Ag35 (dominant isotope from [M + 2H]^2+^) (*m*/*z* 1,022.60) shown in Fig. 6C (see also Fig. S8 and S10 and Table S5). Britacidin A (3) has an exact mass of 2,042.1757 and shares structural similarities with gramicidin A (see comparison in Fig. 7A). Through a combination of fragmentation analyses and ^13^C-labeling experiments, we determined that britacidin A differs from gramicidin A in three key ways. (i) At the first position, the dominant amino acid is isoleucine rather than valine (Fig. 6B and 7A; see also Fig. S15). (ii) Britacidin contains additional valine and alanine residues incorporated at positions 7 and 8 (Fig. 6A and 7A; see also Fig. S11). (iii) At position 11, britacidin contains tyrosine as the dominant amino acid, a position that invariably incorporates a tryptophan in gramicidins (Fig. 6A and 7A; see also Fig. S11). We identified two other analogs, termed britacidin B (4) and britacidin C (5), also made by B. brevis *in vitro* (Fig. 6A; see also Fig. S12 and S13). We identified the MIC of a mixture of britacidins to be 81 μg/ml using a liquid dilution method against Bacillus subtilis 168 (see supplemental methods at https://doi.org/10.6084/m9.figshare.12107094). Taken together, these data indicate that B. brevis produced a known antimicrobial (tyrocidine A) and a novel gramicidin-family antimicrobial (britacidin A) *in planta*.

### The britacidin biosynthetic gene cluster.

To identify a putative gene cluster responsible for production of the britacidins, we sequenced the genome of B. brevis Ag35 (NCBI accession no. JAAKZO000000000) and annotated the biosynthetic gene clusters using AntiSMASH 5.0 (75). We identified the presence of 11 putative biosynthetic gene clusters, including 1 that matched a typical tyrocidine gene cluster with 81% similarity and a gramicidin-like gene cluster with 91% similarity. We focused on the gramicidin-like gene cluster, which we propose to be responsible for britacidin production; thus, we term them the *bri* genes (Fig. 7A and B). Within nonribosomal peptide synthetase (NRPS) enzymes, the 10 amino acid residues that surround the binding pocket within the adenylation (A) or AMP-binding domains determine which amino acid is added to the growing peptide. There are several key differences between the canonical gramicidin gene cluster harbored by B. brevis ATCC 8182 and the putative britacidin gene cluster harbored by B. brevis Ag35. First, two additional AMP-binding domains were found in *briB*, the second predicted NRPS gene in the cluster (Fig. 7B). These additional A domains are predicted to incorporate the extra alanine and valine residues (Fig. 7B and C) at positions 7 and 8 (Fig. 7A). These additional domains and predictions correlate directly with the structure determined for the britacidins described in the Fig. 6A and Fig. 7A legends. The residues that define the binding pocket in A domains can be condensed into a sequence, known as the Stachelhaus sequence (76), which can be used to predict the identity of amino acids incorporated by NRPS enzymes and to compare A domains. Another key difference between the canonical gramicidin gene cluster and the putative britacidin gene cluster lies in the Stachelhaus sequence of the A domain that determines the amino acid found in position 11 of britacidin, which we propose to incorporate a tyrosine residue based on structural elucidation of britacidin A to C. In the gramicidins, the corresponding residue is almost always a tryptophan. Accordingly, we note that the Stachelhaus sequence of the corresponding A domain in the britacidin cluster differs significantly from the Stachelhaus sequence of the corresponding position in the canonical gramicidin cluster (Fig. 7C; see also Fig. S14). For position 13, the amino acid differs in both the gramicidins and britacidins and the Stachelhaus sequences for this position are nearly identical across the two (Fig. 7A and C; see also Fig. S14). Overall, the novel structural features of the britacidins are accounted for by the unique variations observed in the *bri* gene cluster harbored in the genome of B. brevis Ag35.

## DISCUSSION

The root nodules of legume plants play host to a relatively simple associated microbiome (18). Several studies have provided examples of microbes isolated from root nodules that produce antimicrobial compounds *in vitro* (18, 63). These observations prompted us to consider legume root nodules as systems that might be (i) ideal for development as simplified experimental microbiomes for exploring interactions mediated by specialized metabolites and (ii) fruitful in terms of novel compound discovery.

Multiple studies have used 16S amplicon sequencing to profile root nodule communities, including those of Medicago sativa (57), Lotus japonicus (58), and Glycine max (68). Across these studies, the major phyla that were consistently observed as nodule associated included *Actinobacteria*, *Proteobacteria*, and *Firmicutes*. The work of Xiao and coworkers has shown this community to be relatively low in diversity compared to the rhizosphere or root endophyte communities (57). In this work, we began with nodules from alfalfa plants from an agricultural field in Alturas, CA. We separated nodules on the basis of whether they were young (small with no pink coloration), active (based on heme/pigmentation presence), or senescent (based on the presence of oxidized leghemoglobin) and asked if the microbiomes differed across these phases.

Not surprisingly, the nodule community was dominated by *Rhizobia* at all phases; however, we found that the progression from young to active to senescent was accompanied by an overall increase in nonrhizobial relative abundance. This result parallels observations made in the 1970s and before that revealed that older nodules tended to have abundant “contaminants” that complicated the straightforward isolation of the symbiotic *Rhizobiales* (77). One possible explanation is that the increased bacterial richness in nodules as they progress toward senescence indicates increased an potential for latent saprotrophy. Alternatively, older nodules may simply have had more time to acquire a diverse microbial community. Across this progression, we also observed an increase in the relative abundance of *Actinobacteria* and TM7, whose members are thought to be broadly associated with *Actinobacteria* as epibionts (78). In contrast, we found that *Proteobacteria* and *Bacteriodetes* showed a general decrease in relative abundance across these stages. Finally, *Firmicutes* bacteria were found to make up a very small fraction of the community but their relative abundance did not fluctuate. Overall, these results indicate that root nodules have a microbial community that is dynamic across root nodule developmental phases; thus, we suggest that future studies take these phases into account when proceeding with isolations or community analyses. Beyond this, we note that the finding that the nodule microbiome shifts with these phases reinforces the conclusions of Edwards and coworkers (79), who showed that a key driver of root endosphere community structure was the developmental stage of the host plant.

To begin characterizing the functional relationships between members of the root nodule microbiome, we sought to develop a simplified, tractable nodule microbiome system that was amenable to experimental manipulation *in planta.* We took an approach similar to that of Niu and coworkers (11) in which the plant itself was used to select a simplified endophyte community. To do so, we started with crushed root nodules from mature agricultural alfalfa plants and applied this inoculum to a gnotobiotic system containing alfalfa seedlings and Sinorhizobium meliloti RM1021. After three rounds of passaging, the accessory nodule community contained just four culturable species, B. brevis, *Paenibacillus* sp., P. agglomerans, and *Pseudomonas* sp. These four bacterial species were present in the original 16S community profiles of the agricultural nodules, although at very low relative abundance. However, after just two rounds of passaging through root nodules, these four bacterial species dominated the community, suggesting that this simple community was the result of relatively strong selection within this system. The selection pressures in such a gnotobiotic system undoubtedly differ from the complex environmental conditions found in the field. One key difference may include a strong pressure to thrive on root exudates in an artificial environment. Additionally, soil structure, soil nutrients, and the continued presence of the surrounding soil microbial community, which are absent in our gnotobiotic system, may influence the microbiome across the nodule life cycle. However, the four bacterial species that comprise this community have also been reported to be associated with root nodules from a variety of legumes (18, 64, 68, 80–82), indicating that these microbes are frequently found in natural root nodule accessory communities.

The simplicity of this community allowed us to reconstruct it in every possible combination *in vitro* and *in planta*. This enabled us to see the impact of each member on the other members of the community, with results that demonstrated complex examples of cooperation and competition among them. For example, *Pseudomonas* sp. Ag54 was not recovered from nodules when inoculated as the sole accessory community member; however, it was frequently recovered when other community members were also included. When *Pseudomonas* sp. Ag54 and *Paenibacillus* sp. Ag47 were coinoculated, *Pseudomonas* sp. Ag54 was recovered from greater than 80% of the nodules. This pairing was also beneficial for *Paenibacillus* sp., whose frequency also increased in the coinoculated nodules. The mutual benefit observed in this interaction is a notable example of *in planta* cooperation among members within this community. We note that while this cooperation was apparent in terms of the recoverability of both organisms, the average abundance (CFU) of *Paenibacillus* sp. Ag47 decreased with the addition of *Pseudomonas* sp. Ag54. These results lead us to hypothesize that relationships between microbes likely shift during different processes or nodule life phases. For example, *Paenibacillus* sp. Ag47 and *Pseudomonas* sp. Ag54 may cooperate during nodule colonization (boosting the recovery rate of each) but may develop a competitive relationship as they persist over time within nodules (having a negative effect on *Paenibacillus* sp. Ag47 average abundance).

Intriguingly, when B. brevis Ag35 was coinoculated with *Pseudomonas* sp. Ag54 and *Paenibacillus* sp. Ag47, both *Pseudomonas* sp. Ag54 and *Paenibacillus* sp. Ag47 showed reduced recovery rates compared to the results seen when they were inoculated as a duo, and B. brevis Ag35 showed reduced recovery compared to the results seen when it was inoculated as the sole accessory community member. This dynamic indicates that the benefit of cooperation between *Pseudomonas* sp. Ag54 and *Paenibacillus* sp. Ag47 was insufficient to counteract the negative effect of competition with B. brevis Ag35. Such cooperative interactions, and their disruption through competition, are ideal for further exploration at the mechanistic level. Beyond the accessory community interactions, it is important to consider how the community members impact the nodule symbiont. As seen in Fig. 3C, each accessory community member appears capable of antagonism against S. meliloti. Therefore, follow-up studies should aim to assess the impact of these members on the rhizobial symbiont *in planta* with a specific focus on assessing how the accessory microbiome may influence total nitrogen fixation.

Recent studies of interactions within synthetic communities based on plant microbiomes found widespread inhibitory interactions and led to the discovery of novel antimicrobial compounds. For example, the THOR model rhizosphere microbiome, presented by Lozano and coworkers (16), was found to contain Pseudomonas koreensis, the producer of koreenceines A to C. These molecules had inhibitory activity against another member, Flavobacterium johnsoniae. In another example, in a phyllosphere model presented by Helfrich and coworkers (15), binary interaction networks were created and bioactivity-guided fractionation led to the discovery of multiple novel molecules produced by *Brevibacillus* sp. Leaf182, including marthiapeptide A and phosphobrevin. Similarly, in our root nodule system, inhibitory interactions were common. B. brevis was particularly notable, as it produced strong antibiotic activity and was capable of inhibiting the growth of every other member. We found that a component of the antibiotic activity produced by B. brevis was attributable to a set of novel gramicidin-family metabolites that we term the britacidins. Structurally, the britacidins differ from typical gramicidins in that (i) they feature an extended peptide backbone that includes additional alanine and valine residues and (ii) they frequently contain tyrosine residues at positions 11 and 13, which are usually tryptophan residues in typical gramicidins. These structural modifications coincided with a putative gene cluster found in the B. brevis genome with extremely high fidelity. This is notable from a biosynthesis standpoint, as the gramicidin NRPS enzymes have recently been a focus of interest from structural, catalytic, and bioengineering perspectives (83, 84). Thus, the britacidin gene cluster may have value as a natural source for alternative functionality within a well-understood enzymatic system.

Brevibacilli have been isolated in many studies aiming to identify plant-growth-promoting-rhizobacteria and are well-known producers of a range of antimicrobial compounds, including the gramicidins and cyclic peptides of the tyrocidine family. Thus, our findings, together with the findings of Helfrich et al. (15), reinforce the idea that B. brevis may be a widespread member of plant microbiomes with a strong capacity for specialized metabolism. In keeping with this notion, the B. brevis strain from this nodule community produced tyrocidine A and B, in addition to the britacidins. We also examined specialized metabolism *in planta* by using high-resolution metabolomics to characterize extracts from root nodules inoculated with our simplified nodule community. This led to the key finding that both the britacidins and tyrocidines were detectable in these extracts, indicating that B. brevis specialized metabolism was active *in planta*.

An untargeted metabolomics strategy, combined with a subtractive analysis pipeline, enabled us to attribute different chemical features to the plant and individual microbes within the simplified nodule community both *in vitro* and *in planta*. In addition to highlighting the britacidins and tyrocidines, this analysis yielded diagnostic molecules for B. brevis, S. meliloti, and M. sativa. We next used high-resolution, subatmospheric MALDI mass spectrometry imaging to assess the spatial distributions of these diagnostic molecules within cross-sectioned nodules. Veličković and coworkers observed metabolic asymmetry in the root nodule, highlighting the spatial complexity that can exist within this system (85). Our imaging mass spectrometry analysis revealed a strong spatial correlation between heme B, likely associated with the leghemoglobin of the nodule, and a chemical feature associated with S. meliloti. The region of high overlapping of these signals likely defines the area where N-fixation occurs. We also observed that a chemical feature associated exclusively with B. brevis was found in a region distinct from the areas defined by heme and S. meliloti signals. We interpret this to mean that B. brevis likely inhabits areas of the nodule outside the region of active N-fixation. Beyond this, a chemical feature which was strictly associated with nodules inoculated with the entire four-member accessory community was observed in yet another region of the nodule distinct from the areas with signals diagnostic of B. brevis or S. meliloti. Together, these results indicate that the members of the nodule community are likely spatially segregated within the nodule. Such segregation may arise from competitive exclusion within this community (86).

In total, the findings presented here indicate that root nodules, dedicated organs for the critical activity of N-fixation, also host a microbiome with members that actively produce antimicrobials. We speculate that production of antimicrobials in the context of the nodule might influence the content and, ultimately, the function of the resident microbiome. Moreover, antimicrobial biosynthesis might also provide protection from pathogens that might infect these organs, thus ensuring that the critical activity of N-fixation is preserved. The simplified root nodule community that we developed here is a tractable system for directly exploring these potential roles for specialized metabolites. Beyond this, the nodule microbiome community may be ideal for pursuing key outstanding issues in the field of microbiome science, such as mechanistic exploration of spatial structuring or evaluation of cooperation and competition in the context of plant microbiomes.

## MATERIALS AND METHODS

### Collection of agricultural Medicago sativa isolates from Alturas, CA.

M. sativa plants were carefully sampled (with roots and rhizosphere intact) at Alturas Ranches, in Alturas, CA. The plants were transported for 6 h at room temperature (RT) and subsequently rinsed with double-distilled water (ddH_2_O) until visible soil particles from rhizosphere were removed.

### Root sterilization and root nodule collection.

The roots were surface sterilized with commercial bleach for 2 min, rinsed with filter-sterilized double-distilled water (ddH_2_O), and then transferred to 70% ethanol for 30 s. Roots were rinsed with sterile ddH_2_O five times, and the content from the last rinse was concentrated and plated on tryptic soy agar (TSA) and International Streptomyces Project (ISP) no. 2 agar to check for full removal of surface bacteria. Surface-sterilized root nodules were cut free from the plant at the base of the nodule using a sterile blade and sorted into three phenotype groups: young (white), active (pink/red), and senescent (dark green) as seen in Fig. 1A. Nodules were pooled in groups of 10 and crushed in microcentrifuge tubes using a sterile pestle gun for 30 s. Crushed nodules were resuspended in 200 μl deionized water for further processing (Fig. 2A).

### Environmental DNA (eDNA) extraction and 16S rRNA amplicon sequencing.

All root nodule samples were processed with a PowerPlant Pro DNA extraction kit (Qiagen). Amplicon sequencing libraries were prepared by amplifying the V3-V4 region as described by Simmons et al. (87) using Q5 Hot Start polymerase master mix. PCR conditions were optimized for low eDNA yield by the following modification: annealing at 60°C for 60 s. Paired-end sequencing was performed on the MiSeq at the QB3 facility (SRA accession no. PRJNA608732). The data were analyzed using the Mothur MiSeq protocol, and clustering was done using the agc method (88) (accessed 10 May 2018) and phyloseq (89) with R v3.5.0 in R studio v1.1.447.

### Passaging experiment.

We sterilized ∼400 seeds by mixing them with ethanol for 30 min, removing the ethanol, and then mixing the seeds with bleach for 30 min. Seeds were copiously rinsed five times with ddH_2_O and then germinated on Jensen’s agar (65) for 3 days at RT away from direct light (covered in foil). Plant microcosms were constructed as described by Jones et al. (65) with modifications: 40 ml of Jensen’s agar was used in 100-by-15-mm plastic petri dishes. Root inocula prepared as follows: 50 μl of crushed root nodule mixture containing young, active, and senescent nodules from Alturas, CA, was diluted in 1 ml of sterile ddH_2_O. S. meliloti at a final optical density at 600 nm (OD_600_) of 0.05 was added at 1:1 to the mixture to ensure formation of nodules. Plants were grown in growth chambers constructed from sterilized germination trays with tall clear propagation domes that were sterilized with UV, bleach, and 70% ethanol prior to being used as a growth chamber. These trays were placed under 16-h light/8-h dark conditions at 25 ± 2°C. Control plants were inoculated with either sterile water or S. meliloti at an OD of ∼0.05. Root nodules were harvested at 21 days and used for the next passaging. Plants were measured, and roots were sterilized using the protocol described above. The use of a total of 10 root nodules per sample × 3 samples × 3 phenotypes × 4 conditions resulted in 36 samples per passage. For each passage, 15 microcosms were constructed containing two plants per microcosm. Harvesting of last passage was conducted at 5 weeks to ensure full development of root nodules, and 10 root nodules per sample × 5 samples × 3 phenotypes × 4 conditions were collected from this passage.

### Bacterial isolates.

Root nodules from all conditions were crushed with a sterile pestle and resuspended in 50 μl sterile ddH_2_O. Postpassaging isolations were performed from 50 μl of a 200-μl root nodule sample suspension. All of the 50-μl samples from each condition and phenotype were pooled, and 10 μl of a 1:2, 1:10, or 1:100 dilution was plated on the following media: International Streptomyces Project 1 (ISP1), ISP2, ISP3, ISP5, ISP7, tryptic soy agar (agar 15 g/liter, casein peptone 15 g/liter, sodium chloride 5 g/liter, and soya peptone 5 g/liter), potato dextrose agar (potato starch 4 g/liter, dextrose 20 g/liter, and agar 15 g/liter), SM3 (dextrose 10 g/liter, peptone 5 g/liter, tryptone 3 g/liter, sodium chloride 5 g/liter, and agar 15 g/liter), and SKM (skim milk 10 g/liter, magnesium sulfate 0.5 g/liter, and Gelzan 8 g/liter).

### Species identification.

Colony PCR was performed on isolates grown on ISP2 or LB for 4 days. One colony was added to 20 μl ddH_2_O and boiled for 10 min at 98°C in a thermocycler. The cell debris was then pelleted by centrifugation, and DNA concentrations were measured with a NanoDrop spectrophotometer. A 60-ng volume of DNA was used as a template for PCR with primers 1492R (5′-GGTTACCTTGTTACGACTT-3′) and 27F (5′-AGAGTTTGATCCTGGCTCAG-3′) to amplify the 16S gene and was subjected to BLAST analysis for genus identification.

### Microcosm experiments with a selected accessory community.

Seeds were sterilized by mixing them with ethanol for 30 min, removing the ethanol, and then mixing the seeds with bleach for 30 min. The seeds were copiously rinsed five times with ddH_2_O and then germinated on Jensen’s agar (65) for 3 days at RT away from direct light (covered in foil). Plant microcosms were constructed as described above. Root inocula prepared as follows. Bacteria were grown on Lennox LB for 24 h, and a colony was subsequently picked and resuspended in ddH_2_O and the OD_600_ taken. Bacteria were added at an OD_600_ of 0.05 in a 1:1 ratio in a final volume of 200 μl per microcosm to ensure that the same numbers of cells of a single bacterium were added across all conditions. Plants were grown in growth chambers constructed from UV-, bleach-, and 70% ethanol-sterilized germination trays with tall clear propagation domes. These trays were placed under 16-h light/8-h dark conditions at 25 ± 2°C. Control plants were inoculated with either sterile water or S. meliloti at an OD of ∼0.05. Root nodules were harvested at 14 days. Plants were measured and roots were sterilized using the protocol described above. Roots were sterilized with bleach for 30 s and with ethanol for 45 s and rinsed 5 times with water, and root nodules were removed with sterile blade and forceps.

### *In vitro* bacterial growth on root nodule medium and chemical extraction.

Each bacterium was grown on Lennox LB agar at 30°C overnight, and single colonies were transferred to 5-ml Lennox LB liquid cultures. Cultures were shaken at 200 rpm overnight at 30°C, the cells were pelleted and washed with ddH_2_O, and the OD_600_ was adjusted to 0.5. All bacteria were spotted at a volume of 1.5 μl in quadruplicate on root nodule medium (5 g/liter malic acid, 1 g/liter Casamino Acids, 0.2 g/liter NaCl, 0.2 g/liter K_2_HPO_4_, 0.2 g/liter MgSO_4_·7H_2_O, 1 m g/liter H_3_BO_3_, 1 m g/liter ZnSO_4_·7H_2_O, 0.5 m g/liter CuSO_4_·5H_2_O, 0.5 m g/liter MnCl_2_·4H_2_O, 1 m g/liter NaMoO_4_·2H_2_O, 0.1 g/liter FeCl_3_·6H_2_O, and 18 g/liter agar, pH 7.5). After 3 days, four plugs were removed from the plate and extracted in methanol (MeOH)-washed 1.5 ml Eppendorf tubes with 500 μl MeOH, sonicated for 5 min, and incubated for 12 h at room temperature. The MeOH was removed, and the reaction mixture was transferred to clean MeOH-washed Eppendorf tubes. Extracts were dried using a SpeedVac at 45°C and stored at −20°C until the time of sample processing.

### Bioactivity agar plug diffusion assay.

Each bacterium was grown on root nodule medium for 3 days in triplicate at 30°C. An agar plug from each plate of bacteria was placed on a fresh lawn of each bacterium and incubated at 30°C overnight. The presence (a clearing) or absence (bacterial growth around the agar plug) of bacteria was recorded.

### LC/HRMS analysis.

Samples were resuspended in a 150-μl volume of LC/MS-grade MeOH/100 nM reserpine solution, sonicated for 10 min, subjected to vortex mixing, and spun down for 10 min at 15,000 rpm to ensure that no particulate was present in the sample. A 100-μl volume was transferred into an insertion and 50 μl transferred to a pooled quality control (QC) mix comprised of all samples. Using a random number generator, samples were analyzed in a random order. Samples were analyzed by the use of an ultra-high-pressure liquid chromatography (UHPLC) system (Dionex Ultimate 3000; Thermo Fisher, USA) coupled to a high-resolution mass spectrometer (HRMS; Thermo Q-Exactive Quadrupole-Orbitrap; Thermo Fisher, USA) using a heated electrospray ionization (HESI) source and a C_18_ column (Thermo Scientific Acclaim rapid-separation liquid chromatography [RSLC] system) (50 mm by 2.1 mm, 2.2-μm pore size). The UHPLC method was as follows: 0 to 1 min 10% acetonitrile (ACN) plus 0.1% formic acid (FA), a gradient of 10 to 11 min of 10% to 98% ACN plus 0.1% FA, a gradient of 110 to 14.5 min of 98% ACN plus 0.1% FA, and reequilibration of the column into 10% ACN plus 0.1% FA from 14.50 to 18 min; injection volume of 5 μl, flow rate of 0.4 ml/min, and column oven temperature of 35°C. The full MS1 scan was performed in positive mode at a resolution of 35,000 FWHM (full width at half-maximum) with an automatic gain control (AGC) target of 1 × 10e6 ions and a maximum ion injection time (IT) of 100 ms with a mass range from *m*/*z* 200 to *m*/*z* 2,000. MS/MS analysis data were acquired using a data-dependent Top5 method at a resolution of 17,500 FWHM with an AGC target of 1 × 10e5 ions and a maximum ion IT of 50 ms, using an isolation window of 3 *m*/*z* and normalized collision energy (NCE) values of 20, 30, and 45. The cone spray voltage was 3.5 kV. Data were processed using MS-DIAL software and analyzed using R v3.6.1 and the data.table R package (90), and Venn diagrams were made using Adobe Illustrator v23.1.1.

### Sample preparation and acquisition of MALDI-IMS.

Fresh roots with root nodules were embedded in gelatin as described previously by Gemperline and Li and were sliced at 20-μm intervals using a cryostat (Leica CM3050 S) at −25°C (73). Slices were transferred to an indium-tin oxide (ITO)-coated microscope slide and stored at −20°C until the samples were ready to be processed. Samples were thawed at room temperature inside a desiccator under vacuum (0.6 MPa) for 30 to 45 min. Micrographs of the root nodule slices were acquired using a Zeiss microscope (Zeiss Axio Zoom v.16 equipped with AxioCam 506 color camera), and a Super-DHB MALDI matrix (Sigma-Aldrich) was deposited on the top of the samples using a sublimation method described previously by Pessotti et al. (38) to achieve a spatial resolution of 10 μm. MALDI-IMS was performed in positive mode using a SubAP/MALDI (nanogram) source (MassTech, Columbia, MD) coupled to a Thermo Q-Exactive HRMS. Full MS1 scans were acquired in positive mode at a resolution of 35,000 FWHM (full width at half-maximum), a mass range of *m*/*z* 100 to 2,000, an AGC target of 1 × 10^6^ ions, and a maximum IT of 400 ms. The pixel size was 10 μm, with a laser velocity of 1.5 mm/min and laser energy of 50% at 1-kHz repetition rate. Imaging processing and analysis were performed using Datacube Explorer v2.3 (91), MSiReader v1.01 (92), and ImageJ v1.52a (93).

### gDNA extraction and sequencing and assembly of Brevibacillus brevis Ag35 genome.

Brevibacillus brevis Ag35 was grown at 30°C with shaking at 200 rpm in 20 ml of Lennox LB for 24 h. Cells were pelleted at 4,000 rpm for 10 min at 4°C. The protocol used was adapted from the recommended Pacific Biosciences genomic DNA (gDNA) cleanup method with modifications. Pellets were washed with a mixture of 10 mM Tris-HCl and 1 mM EDTA (pH 8.0). Cells were resuspended in a mixture of 740 μl 10 mM Tris-HCl and 1 mM EDTA (pH 8.0), treated with 2 mg/ml lysozyme, and incubated for 30 min. A 40-μl volume of 10% (wt/vol) SDS mixed with 10 μl proteinase K was added, and the solution was incubated at 55°C for 60 min. After the solution cleared, 100 μl 5 M NaCl was added and the solution was incubated at 65°C for 10 min. The solution was then cooled on ice, and then a 1:1 volume of chloroform/isoamyl alcohol (24:1) was added. The solution was inverted 20 times, and the phases were separated by centrifugation at 5,000 rpm for 10 min. The top phase was transferred to a new tube, and a volume of phenol/chloroform/isoamyl alcohol (25:24:1) (pH 8.0) was then added at 1:1. The solution was inverted, and the phases were separated by centrifugation at 500 rpm for 10 min. The top phase was transferred, and the 1:1 chloroform/isoamyl alcohol (24:1) step with centrifugation was repeated. The top phase was transferred, and gDNA was fished out using a clean p1000 pipette tip and transferred to a fresh tube. The gDNA was washed with ice-cold isopropanol followed by ice-cold ethanol. Genomic DNA was then resuspended in 10 mM Tris-HCl–1 mM EDTA (pH 8.0) and was treated with RNase at 37°C for 15 min followed by the addition of a 1:10 volume of 3 M sodium acetate. Two volumes of cold ethanol were then added to precipitate the DNA, which was then was fished out using a clean p1000 pipette tip and transferred to a fresh tube. The DNA was washed with 75% ethanol and air dried. The DNA was sequenced using PacBIO technologies. Sequencing data were partitioned using seqtk followed by genome assembly performed with Flye at 222× coverage. Each genome was annotated using AntiSMASH 5.0.

### Purification and antimicrobial activity of britacidins.

B. brevis Ag35 was grown on ISP2 agar for 48 h and extracted 2:1 with EtAc. Size exclusion chromatography was performed using a 1.5-cm diameter glass column with Sephadex LH-20 resin swelled in methanol for 3 h at room temperature (18 g of resin with 72 ml of methanol). The column was packed under gravity conditions to achieve a final bed height of 41 cm. The column was equilibrated with 2 column volumes (CV) of methanol. The dry sample (27.5 mg) was resuspended in a 1.4-ml volume, sonicated, and then centrifuged for 2 min at 10,000 × *g*, and the supernatant was loaded onto the column. Eluents were collected under gravity flow. The first 32-ml volume was discarded. The next 12-ml volume was collected and run on LC/HRMS to confirm purity.

### Structural elucidation of britacidins.

The unknown feature *m*/*z* 1,033.0829 detected in the root nodules was also detected in B. brevis cultures. Five other features were observed in the same MS1 spectrum: *m*/*z* 1,022.0960, 1,033.0848, 1,044.0746, 2,043.1830, and 2,065.1664. The isotopic pattern and MS2 spectra suggested that these five features represent different adducts of the same compound, specifically, *m*/*z* 1,022.0960 = [M + 2H]^2+^, *m*/*z* 1,033.0848 = [M+H+Na]^2+^, *m*/*z* 1,044.0746 = [M + 2Na]^2+^, *m*/*z* 2,043.1830 = [M+H]^+^, and *m*/*z* 2,065.1664 = [M+Na]^+^. Database searches did not provide any hits with known molecules. The observed fragmentation pattern proved to be very similar to the fragmentation pattern of the analytical standard of gramicidin A obtained from B. brevis (Sigma-Aldrich) (see Fig. S9 at https://doi.org/10.6084/m9.figshare.12107094). Therefore, it was hypothesized that this unknown feature represents a new analog of the gramicidin family.

In order to investigate the structure of this potentially novel compound, we used a low normalized collision energy (NCE) value (NCE = 15) that allowed us to see the amino acids losses of the [M + 2H]^2+^ and [M+H]^+^ adducts. The largest value representing the fragment that was detected in common between gramicidin A and the unknown feature was *m*/*z* 959.5511, which corresponds to the gramicidin A protonated *Y*_6_ product ion (C_53_H_71_N_10_O_7_^+^, with a measured error of 0.94 ppm; see Fig. S9 and S11), which suggests that this compound and gramicidin A differ structurally at position 1 to position 13 (Fig. 7A; see also Fig. S9). The *b* product ion series was found to be of higher abundance than the *Y* series and was therefore chosen to follow the successive amino acid losses to elucidate the identity of the unknown molecule. Table S11 (https://doi.org/10.6084/m9.figshare.12107094) shows all the detected amino acid loss data and summarizes the suggested chemical formula and parts-per-million (ppm) error of each predicted ion of both the *b* and *y* series.

The fragmentation pattern revealed that, compared to gramicidin A, the unknown compound, termed britacidin A (Fig. 7A), has two additional alanine and valine residues at positions 7 and 8 and a tyrosine at position 11 instead of a tryptophan. The common substitution from valine to isoleucine in the gramicidin family at the first amino acid position (74) was observed; fragments *m*/*z* 114.09 and 142.08 corresponded to the *a*_1_ and *x*_1_ product ions, respectively, that resulted from the fragmentation that occurred between the glycine residue at position 2 and isoleucine residue at position 2 (Fig. 7A). Because isoleucine and leucine are the same molecular weight, we performed [^13^C]isoleucine and [^13^C]leucine feeding experiments (see supplemental methods at https://doi.org/10.6084/m9.figshare.12107094) to confirm that the amino acid at position 1 was isoleucine (see Fig. S15).

Other britacidin analogs were observed, but only two of them were abundant enough to allow structural elucidation using fragmentation patterns. These analogs were named britacidin B and C (Fig. 6A; see also Table S12 and S13).
